# Interactions of β-Lactoglobulin with Bovine Submaxillary Mucin vs. Porcine Gastric Mucin: The Role of Hydrophobic and Hydrophilic Residues as Studied by Fluorescence Spectroscopy

**DOI:** 10.3390/molecules26226799

**Published:** 2021-11-10

**Authors:** Hilal Yılmaz, Seunghwan Lee, Ioannis S. Chronakis

**Affiliations:** 1 Department of Biotechnology, Bartın University, Kutlubey Campus, Bartın 74100, Turkey; 2 Department of Mechanical Engineering, Technical University of Denmark, DK-2800 Kgs. Lyngby, Denmark; seunghwan.lee.jr@gmail.com; 3 DTU-Food, Technical University of Denmark, Kemitorvet, B202, DK-2800 Kgs. Lyngby, Denmark

**Keywords:** fluorescence, β-lactoglobulin, bovine submaxillary mucin, porcine gastric mucin, pH

## Abstract

The aim of this study was to investigate binding interactions between β-lactoglobulin (BLG) and two different mucins, bovine submaxillary mucins (BSM) and porcine gastric mucin (PGM), using intrinsic and extrinsic fluorescence spectroscopies. Intrinsic fluorescence spectra showed an enhanced decrease of fluorescence intensity of BLG at all pH conditions when BLG was mixed with PGM rather than with BSM. We propose that, unlike BSM, the tertiary structure of PGM changes and the hydrophobic regions are exposed at pH 3 due to protonation of negatively charged residues. Results suggest that PGM also facilitated the structural unfolding of BLG and its binding with PGM by a hydrophobic interaction, especially at acidic pH, which was further supported by extrinsic fluorescence spectroscopy. Hydrophobic interaction is suggested as the dominant interaction mechanism between BLG and PGM at pH 3, whereas electrostatic interaction is the dominant one between BLG and BSM.

## 1. Introduction

Proteins are important ingredients for food products to provide desirable textural, sensory, and nutritional properties. To optimize the utilization of proteins in food science and engineering, an in-depth understanding is needed of the various interrelated parameters that influence aggregation, adsorption, and structural behavior of proteins. For instance, the interactions of β-lactoglobulin (BLG), a major whey protein, and mucins, as the major macromolecular component in saliva or gastric fluids, are drawing increasing attentions in the context of understanding the oral processing or digestion of dairy food on the molecular level. Bovine BLG has been one of the most extensively studied proteins, [1,2,3,4,5] mainly due to its abundance in cow’s milk. The concentration of BLG in milk is about 0.2 g/100 mL, which is second highest after casein (2.9 g/100 mL) [6]. BLG molecules exist mainly as dimers at neutral pH and room temperature, whereas they dissociate into monomers at pH < 3, and partly exist as octamers at its isoelectric point (pI~5.2) [7,8]. BLG has a nonpolar interior region and two Tryptophan (Trp) residues, [9] i.e., Trp-19, which is located in the more hydrophobic environment at the bottom of the calyx formed by the antiparallel β-strands, while Trp-61 is positioned adjacent to the strand involved in antiparallel interaction of the dimer [10]. Extensive studies regarding the structure and functions of BLG have provided important information about pH-dependent conformational transitions [11] and binding with various hydrophobic and amphiphilic ligands [9].

Mucin, on the other hand, is one of the major proteins in saliva or gastric fluids, which accompany all food bolus in digestion throughout the oral and gastrointestinal organs. Mucins from different organs have some common features, such as a high degree of glycosylation, high molecular weights ranging from 0.5 to 40 MDa amphiphilic character, and low isoelectric points, estimated to be between 2 and 3 [12,13]. Meanwhile, different mucins with different origins show important differences in their composition, structure, and biophysical properties too. These differences are reported to have originated from both hydrophobic terminal domains, as well as the central regions, where the type, amount, and position of negatively charged moieties are different [12,14,15].

Our recent spectroscopy study based on dynamic light scattering (DLS), circular dichroism (CD) spectroscopy, and nuclear magnetic resonance (NMR) spectroscopy suggested pH-dependent hydrophilic interactions between BLG and mucin [4]. However, there has been no study specifically designed to probe hydrophobic interaction between the BLG and mucins or any role of hydrophobic residues of these proteins yet. In this context, fluorescence spectroscopy can be considered as an optimal technical approach to investigate the hydrophobic interaction between proteins. Fluorescence spectroscopy is based on the presence of intrinsic fluorophores, e.g., Trp residues, in which the emission spectra (both intensity and the maximum peak positions (λ_max_)) are sensitive to the local environment of the Trp residues, and can thus provide useful information on both conformational and structural changes of proteins [16,17,18]. Hence, the changes in the intrinsic Trp fluorescence of Trp-61 and Trp-19 in BLG [1,19,20] while interacting with mucins may provide valuable information on their binding patterns/mechanisms where Trp resides are involved. Furthermore, extrinsic fluorescence spectroscopy, e.g., by employing aromatic fluorescent dyes, such as 8-anilino-1-naphthalene sulfonate (ANS), is broadly used to monitor the conformational changes of proteins [5,21], especially for those that lack strong intrinsic fluorophores. ANS shows particular affinity to hydrophobic domains [22] of native and partially unfolded proteins [23]. Conformational transitions, binding properties, pH and temperature dependence of BLG have been studied for years [5,17,24,25,26,27]. There are, however, only a few fluorescence studies (both intrinsic and extrinsic) available for mucins [28,29]. Moreover, no literature information is available for the interaction of BLG and mucin specifically based on fluorescence technique to date.

Thus, the objectives of the present study were to elucidate the interaction mechanisms of BLG and two types of mucins, namely bovine submaxillary mucin (BSM) and porcine gastric mucin (PGM), specifically focusing on the role of hydrophobic residues of the proteins at different pH conditions. Understanding the molecular level interaction between BLG and mucins may provide a basis for understanding the digestion process of food as influenced by saliva and gastrointestinal fluids. Intrinsic fluorescence spectroscopy and the fluorescent dye ANS techniques were used to assay binding affinity and conformational modifications of the proteins.

## 2. Results and Discussion

### 2.1. Effect of Mucins (BSM and PGM) and pH on the Intrinsic Fluorescence Intensity of BLG

**Figure 1:**
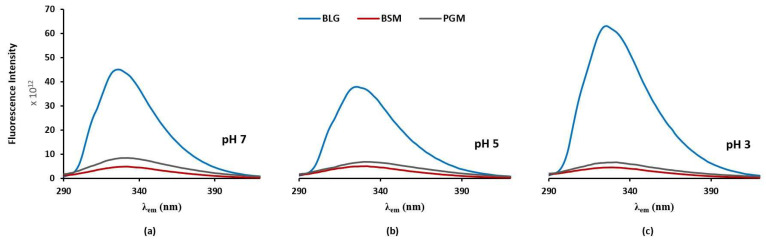
Fluorescence emission spectra (at λ_ex_ 280 nm) of β-lactoglobulin (BLG), bovine submaxillary mucin (BSM), and porcine gastric mucin (PGM) at the same concentration (0.5 mg/mL) in different pH values of phosphate buffer solutions ((**a**) pH 7, (**b**) pH 5, and (**c**) pH 3).

The intrinsic fluorescence emission spectra measured at the same concentration (0.5 mg/mL) for all protein solutions (BLG, BSM, and PGM), are shown in Figure 1 (i.e., excitation at 280 nm and emission in 290–420 nm). The intensities of the emission fluorescence spectra for the two mucins were relatively weaker compared to BLG and nearly independent of pH. This is presumably due to that there are very few Trp residues in the mucins and the numbers of mucin molecules were also small at the same concentration due to their large molecular weights.

The observed λ_max_ for BLG (λ_em_ = 326) is somewhat shorter than the λ_max_ for the indole group of tryptophan alone (λ_em_ = 340 nm), which indicates that the main fluorescing residue of BLG is effectively shielded from aqueous solvent. As compatible with literature, the main fluorescing residue of BLG is Trp-19, which is located in the more hydrophobic environment of the protein molecule [19]. Therefore, any changes on λ_max_ and fluorescence intensity of BLG in the presence of mucin is an indication that BLG-mucin interactions involve the hydrophobic regions of BLG. On the other hand, the observed λ_max_ for mucin (λ_em_ = 350 nm) is slightly longer than the λ_max_ for the indole group of tryptophan alone (λ_em_ = 340 nm), which indicates that the fluorescing residue is located on the surface of the protein in contact with solvent molecules [16]. This is another reason why the mucin molecules had relatively weaker fluorescence intensity compared to the BLG (Figure 1).

The intensity of the intrinsic fluorescence emission signals of the BLG solution (0.5 mg/mL) at its maximum wavelength (λ_max_) were changing with varying pH, and the trend is pH 3 > pH 7 > pH 5 (Figure 1). On the other hand, as pH did not alter the location of λ_max_ considerably, it can be assumed that the pH changes in the environment do not affect the fluorescent tryptophan residue of BLG significantly.

At pH 3, even though BLG is overall positively charged, it becomes more hydrophobic in the sense that nonpolar solutes are more effectively shielded from aqueous solvents and a more compact configuration is formulated [30]. Hence, BLG shows a higher fluorescence intensity at pH 3 due to the preserved Trp residues within “hydrophobic pockets”. BLG at pH 5 is almost uncharged (the isoelectric point of BLG is approximately 5) [8] and Trp residues have a higher chance to be exposed to the solvent. This can account for the reduced fluorescence intensity of BLG at pH 5 compared to the fluorescence intensities at pH 3 and pH 7. Meanwhile, pH dependent changes in the fluorescence intensity of BLG can be explained by monomer-dimer transitions too. At close to pH 3, BLGs are known to be mainly monomeric, yet mainly dimeric at a neutral pH [1]. Mills and Creamer [31] reported that Trp fluorescence of BLG was reduced in intensity by association of the monomeric proteins into dimers, which is consistent with the trend shown in Figure 1.

**Figure 2:**
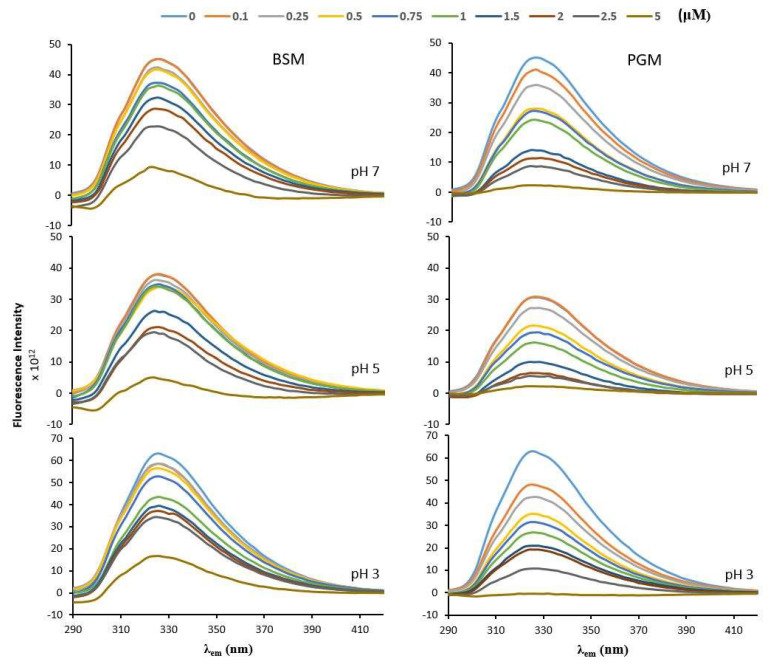
Fluorescence emission spectra (at λ_ex_ 280 nm) of β-lactoglobulin (28 μM) in the presence of increasing concentration of bovine submaxillary mucin (BSM) and porcine gastric mucin (PGM) in phosphate buffer solution with different pH values (pH 7, pH 5, pH 3). Each curve represents a triplicate assay after correction for mucin fluorescence.

The insignificant fluorescence intensity of mucins compared to the BLG enables us to use mucin molecules as a quencher of the intrinsic Trp fluorescence in BLG in the context of investigating the binding interactions between BLG and mucin. To this aim, first, the reference fluorescence intensity, F_0_, was measured for BLG without the quencher (mucins—BSM or PGM), and then the fluorescence intensity, F, was measured at different quencher concentrations but at a fixed total solute concentration in all cases studied. The experimental values are reproducible within 5% of experimental errors. As shown in Figure 2, the addition of increasing concentrations of mucins caused a gradual decrease in the BLG fluorescence intensity.

**Figure 3:**
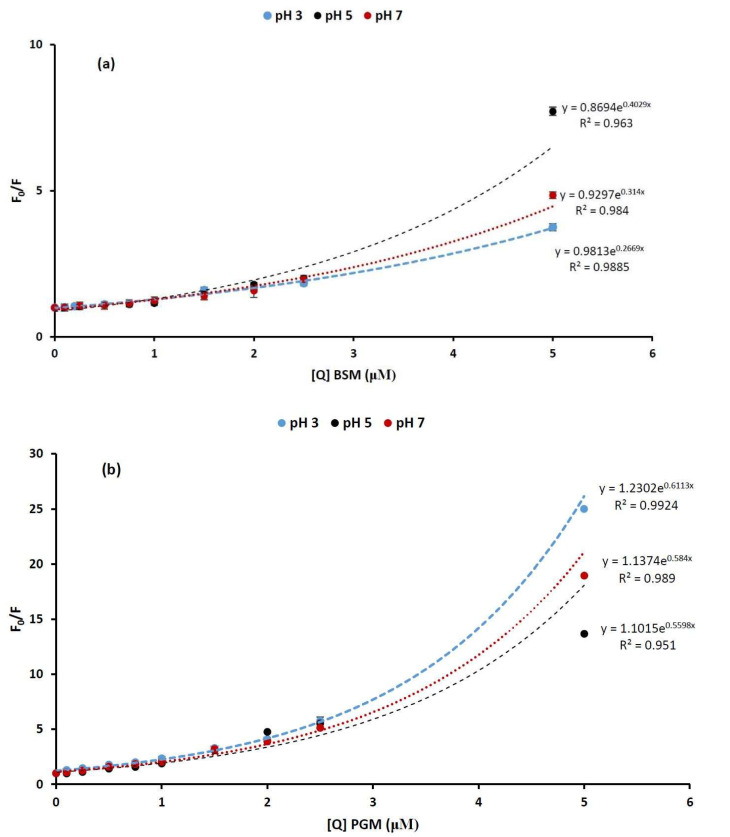
Stern–Volmer plots describing tryptophan quenching of β-lactoglobulin (28 μM) by increasing concentration of (**a**) bovine submaxillary mucin (BSM) and (**b**) porcine gastric mucin (PGM) in different pH values of phosphate buffer solutions (blue: pH = 3, black: pH = 5, red: pH = 7). The fluorescence emission intensity was recorded at λ_ex_ 280 nm and the λ_em_ maximum occurred at 326 nm.

In order to describe BLG quenching by increasing concentration of mucins in different pH conditions, the Stern–Volmer plots, presented in Figure 3, were obtained using the experimentally measured values of F and F_0_. The Stern–Volmer plots are found to be nonlinear within 0–5 μM concentration range of the quencher, showing an upward curvature toward the y-axis at high mucin concentration. Positive deviations from the Stern–Volmer equation (upward-curving plots) are frequently detected when the extent of quenching is large [28]. Such type of deviation from the Stern–Volmer plot is attributed to the static quenching process and generally explained by the “sphere of action” model [16,32]. According to this model, the static quenching takes place if the quencher being adjacent to the fluorophore just at the moment of its excitation. We also assume that at a high mucin concentration, the static quenching occurs, as the quencher molecules (mucin) are very near to the fluorescent molecules (BLG) at the exact moment that they happen to be excited.

**Figure 4:**
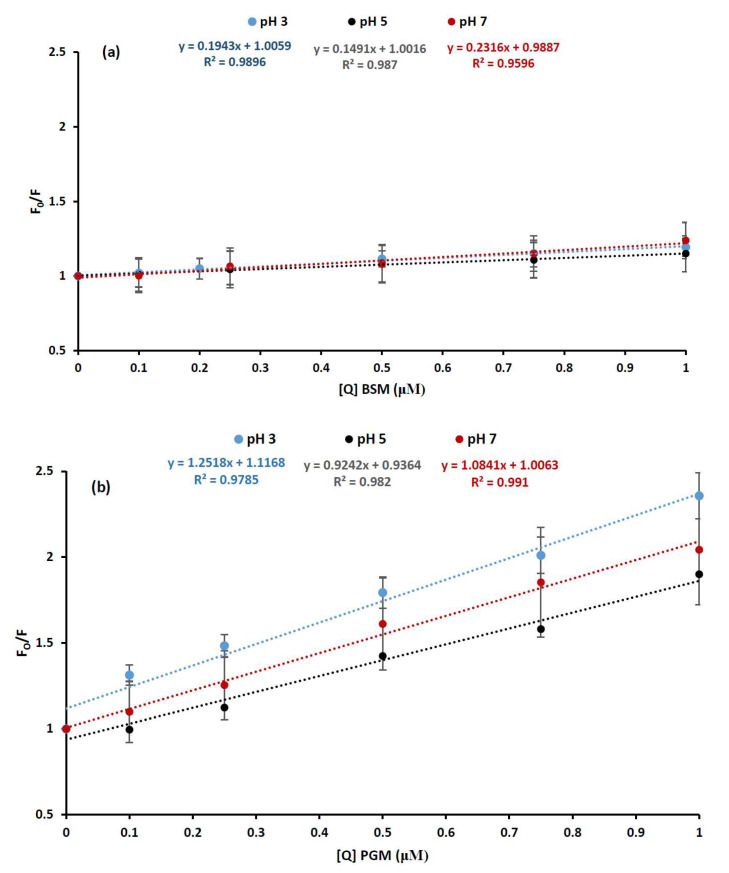
A linear Stern–Volmer relationship describing tryptophan quenching of β-Lactoglobulin (28 μM) at low quencher (mucin) concentrations (≤ 1 μM) in different pH values of phosphate buffer solutions (blue: pH = 3, black: pH = 5, red: pH = 7): (**a**) bovine submaxillary mucin (BSM) and (**b**) porcine gastric mucin (PGM). The fluorescence emission intensity was recorded at λ_ex_ 280 nm and the λ_em_ maximum occurred at 326 nm.

Despite the overall positive deviations, it is also notable that there is a linear region in the Stern–Volmer plots, up to 1 μM concentration of mucins at all pH values, and upward-curving Stern–Volmer plots with a concave shape toward the y axis is observed at a higher mucin concentration only (>1 μM) (Figure 3a,b). The linear section of the Stern–Volmer plots are shown in Figure 4.

The linearity of Stern–Volmer plot (*F*_0_/*F*) versus the quencher concentration ([Q]) generally indicates that one type of quenching mechanism is dominant, either static or dynamic. The fluorescence quenching data were analyzed using Stern–Volmer equation (Equation (1)):

*F*_0_/*F* = 1 + *k_q_τ*_0_[*Q*] = 1 + *K_SV_* [*Q*] (1)

where *F*_0_ and *F* denotes the fluorescence intensity before and after the quencher (mucin) addition, respectively, *k_q_* is the bimolecular quenching constant, *τ_0_* is the lifetime of the fluorophore in the absence of quencher, [*Q*] is the quencher concentration and *K_SV_* is the Stern–Volmer constant, defining the quenching efficiency. The Stern–Volmer quenching constant *K_SV_* for BLG-mucin system was obtained by the slope of the regression curves of *F*_0_/*F* against [*Q*] in the linear range (Figure 4) and written as *K_SV_ = k_q_τ*_0_ [33], where *τ_0_* was the lifetime of the BLG in the absence of the quencher and equaled 10^−8^ s [20]. *K_SV_* and *k_q_* (calculated by using Equation (1)) reflected the efficiency of quenching or the accessibility of the fluorophore to the quencher, and were indicative of the strength of the interaction between BLG and mucins. The calculated *K_q_* values from the linear Stern–Volmer curve equation were 1.943 × 10^13^ (pH 3), 1.491 × 10^13^ (pH 5), and 2.316 × 10^13^ (pH 7) for BSM and 12.518 × 10^13^ (pH 3), 9.242 × 10^13^ (pH 5), and 10.841 × 10^13^ (pH 7) for PGM. All of the calculated *K_q_* values, for either BSM or PGM, were at least two orders of magnitude higher than the limiting diffusion rate constant of the biomolecules (2 × 10^10^ M^−1^ s^−1^). This indicates that the obtained values for bimolecular quenching constants of BLG-mucin systems were higher than the maximum value possible for diffusion-limited quenching (dynamic mechanism) [20,28]. In turn, this means that static, and not dynamic, quenching was the main quenching mechanism between BLG and mucins. In other words, mucins are expected to interact with BLG by a static type, involving the formation of a stable ground-state complex between two compounds, independently of the mucin type and solvent pH.

**Figure 5:**
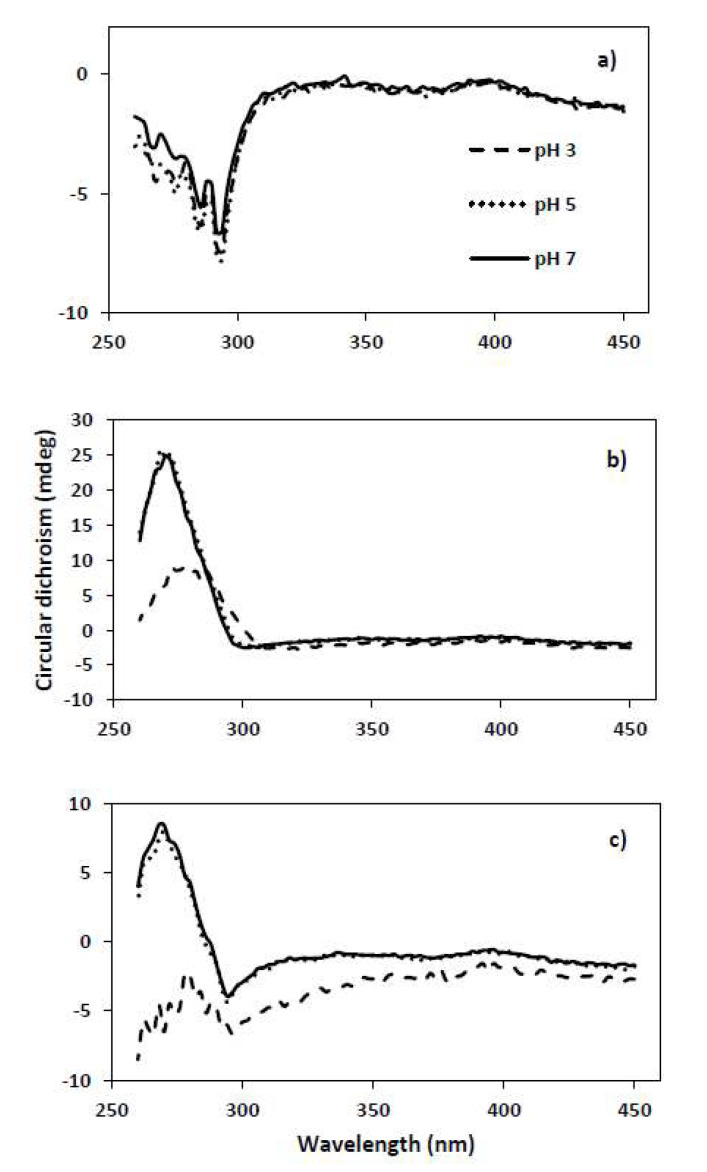
Near-UV CD spectra of (**a**) BLG, (**b**) PGM, (**c**) BLG–PGM mixture at pH 3, 5, and 7.

However, the obtained *K_sv_*, indicating the affinity of BLG to interact with mucin, was observed to have, in fact, a high dependence on mucin type. Higher values in *K_sv_* were observed in the presence of PGM (1.25, 0.92, and 1.08 at pH 3, 5, and 7, respectively) than BSM (0.19, 0.15, and 0.23 at pH 3, 5, and 7, respectively) at all pH conditions. The higher values of *K_sv_* observed from PGM indicate a stronger binding affinity of BLG to PGM than BSM, and a lower concentration of PGM is needed to quench the fluorescence of BLG. In addition, mixing of BLG with PGM led to shifts of the λ_max_ (from 326 to 324 nm when the PGM concentration is higher than 1 μM at all pH values), suggesting that PGM facilitated the structural unfolding of BLG and the binding to PGM by a hydrophobic interaction. Structural changes in the course of interaction between BLG and PGM were also observed by near-UV CD spectroscopy (Figure 5). The tertiary structure of BLG changed significantly when mixed with PGM at pH 3, even though no significant acid-induced changes in tertiary structure of BLG were detected (Figure 5). In contrast, no interaction effect was observed in the near-UV CD spectra of the BLG–BSM mixture [4].

### 2.2. ANS Binding of BLG, BSM, and PGM and Effect of pH on Their ANS Fluorescence Intensity

**Figure 6:**
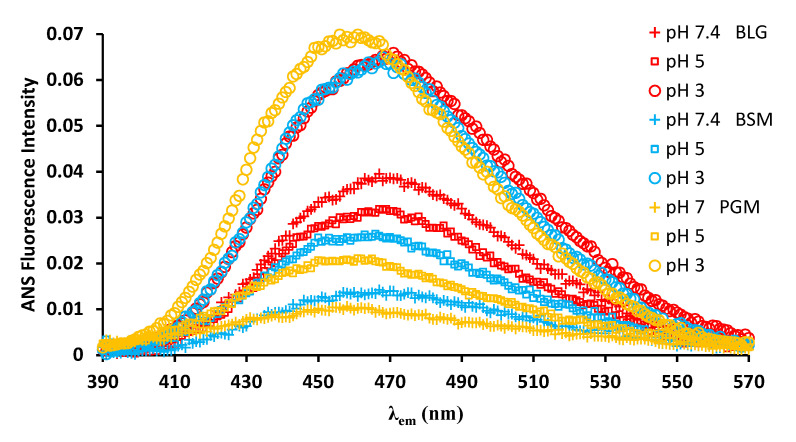
pH dependent fluorescence emission spectra of ANS binding proteins (BLG, BSM, PGM) at pH 7.4, pH 5, and pH 3. (Excitation wavelength: 365.5 nm).

Extrinsic dye ANS-based fluorescence is independent of the presence and position of a fluorophore within the target proteins; hence, it is a valuable addition to understanding the interaction behavior between BLG and mucins. Moreover, minor changes in surface hydrophobicity of the proteins can be monitored by ANS fluorescence, which are not necessarily probed by the tryptophan and tyrosine fluorescence method [23]. In Figure 6, emission fluorescence spectra of ANS in the presence of BLG, BSM, and PGM acquired at different pH values are presented. The spectra shown in Figure 6 were obtained by subtracting the corresponding spectra of the solvent (ANS in respective buffer) at each pH. Emission fluorescence spectra of ANS in an aqueous solvent alone were nearly featureless (data not shown) as it is quickly quenched when directly exposed to the aqueous solvent. However, the quenching can be suppressed in the presence of amphiphilic molecules, such as proteins, if hydrophobic interactions result in shielding of ANS from exposure to the aqueous solvent [23]. In this process, the conformation of the neighboring amphiphilic molecules is a determining factor, which is, in turn, highly dependent on the solvent pH. At pH 7, among the three types of proteins, BLG showed the highest enhancement of fluorescence intensity of ANS, where the fluorescence intensity increased by 76% in the presence of BLG, whereas it increased only by 31% and 21% when accompanied by BSM and PGM, respectively. At pH 5, ANS–BLG slightly lost its fluorescence compared to that at pH 7, whereas both ANS–BSM and ANS–PGM increased the fluorescence intensity compared to at pH 7. Nevertheless, the fluorescence intensity of ANS–BLG was still higher than those of the ANS-mucin solutions. At pH 3, the fluorescence intensity increased most substantially for all the ANS-proteins samples, although the difference in the relative intensities between the different ANS-protein samples was negligible. Lastly, λ_max_ of the three different ANS-protein spectra were in the order of PGM < BSM < BLG with increasing emission wavelengths. It is well known that gastric mucin undergoes a conformational change from a random coil conformation at pH ≥ 4 to an anisotropic, extended conformation at pH < 4 where mucin molecules cluster together [29,34]. The increased ANS fluorescence intensity with decreasing pH, therefore, indicates an increased binding of the hydrophobic ANS molecules to the mucins due to conformational transitions, accompanied by increased exposure of hydrophobic binding sites at the mucins [35].

ANS binding to BLG is stronger at pH 3 than at pH 7 and the minimum value is observed near the isoelectric point (pH 5.2), which is in agreement with previous studies in the literature [24,25]. In other words, the intensity of ANS fluorescence increased as the pH shifted away from the isoelectric point (IEP) of the BLG. Mucins (pI~2), however, have decreasing fluorescence intensity as the pH is shifted away from the IEP (with increasing pH). The reason is that the increasing electronegative charges of mucins with increasing pH (the highest negative zeta potential of mucin at pH 7 has been previously shown) [4] causes a strong repelling effect to the negatively charged probe ANS. Generally, higher affinity of ANS to bind positively charged proteins via electrostatic attraction has been observed in previous studies [26]. Similarly, higher ANS fluorescence intensity of BLG at pH 3 compared to pH 7 is due to the electrostatic attraction, i.e., positively charged BLG at pH 3 is more accessible, and binds to the negatively charged probe ANS. Furthermore, the changes in the magnitude of binding affinity with pH can be attributed to the fact that the conformation of BLG changes with pH. BLG as a globular protein is predominantly in a dimer form around pH 7, but in a monomeric form around pH 3. Since the protein molecules interact with themselves and form dimers, it is possible that some of the binding sites on BLG molecules are hidden at pH 7 [36].

### 2.3. Effect of pH and BLG–Mucin Interaction on ANS Binding and ANS Fluorescence Intensity

ANS fluorescence spectra of the BLG–mucin mixtures are more complicated as they may reflect not only the interaction of ANS with BLG or the mucins, respectively, but also the interaction between BLG and the mucins or their aggregates. In order to analyze the ANS fluorescence spectra of the BLG–mucin mixtures, the measured intensities of the mixtures (i.e., ANS in BLG + mucin mixture solution) were compared with the numerical sum of ANS fluorescence intensities of the two respective proteins (i.e., ANS in BLG solution + ANS in mucin solution). The BLG-mucin mixtures had 1 mg/mL concentration in total and each protein had 0.5 mg/mL concentration. Therefore, an observed decrease in the signal intensity of ANS in the mixtures compared to in the neat BLG (1 mg/mL) solution could simply be a concentration effect. To clarify this, a simple calculation based on fluorescence intensity (FI) was carried out (Equation (2)):

(2) $$\mathrm{Average}\;\left(\mathrm{concentration}\;\mathrm{based}\right)\;\mathrm{signal}\;\mathrm{intensity}\;=\frac{\mathrm{FI}\;\mathrm{of}\;\mathrm{Protein}\;1}{2}+\frac{\mathrm{FI}\;\mathrm{of}\;\mathrm{Protein}\;2}{2}\;$$

**Figure 7:**
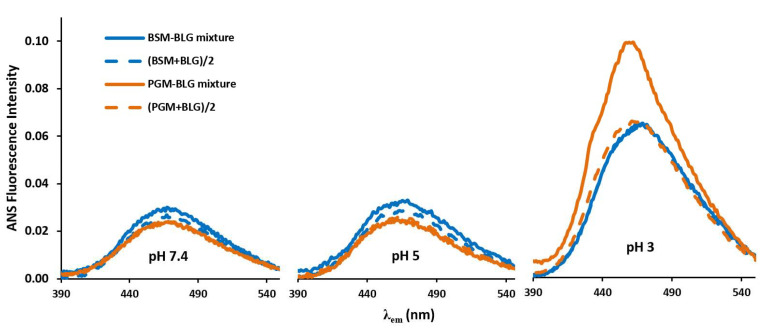
The comparison of the extrinsic fluorescence intensity of the BSM–BLG and PGM–BLG mixtures with their calculated spectra (mucin + BLG)/2 using the spectra of pure BLG and mucins.

These numerical averages of the signals were then compared with the measured values (Figure 7). Thus, if there is no interaction between two proteins, i.e., BLG and mucin, the experimentally determined spectra should be overlapped with the calculated ones, as shown in a previous study by the authors on NMR and circular dichroism (CD) spectroscopy [4].

At pH 5 and pH 7, the two spectra were similar to each other and nearly overlapped for both BLG-mucin mixtures (Figure 7). This trend was also observed in the BLG–BSM mixture at pH 3. However, for the BLG–PGM mixture at pH 3, the experimentally determined ANS fluorescence intensity was much higher than the numerical average of the two spectra. In other words, compared to when ANS was exposed to either BLG or PGM molecules alone, there was a synergetic effect between BLG and PGM in the binding affinity with ANS. This is correlated to the strong associative interaction of BLG with PGM at pH 3. Moreover, it is also noted that the λ_max_ of the PGM–BLG mixture at pH 3 (experimentally determined spectra) showed a shift to a shorter wavelength (i.e., blue shift) compared to the numerical average of the two spectra and other BLG–mucin mixtures (PGM–BLG mixtures at pH 5 and pH 7 and BSM–BLG mixtures at each pH values) as probed by ANS. This effect can be interpreted as further support for the synergetic hydrophobic interaction of the mixture of BLG and PGM at pH 3. It should be also noted that electrostatic attraction of the proteins with ANS might be a contributing factor to the higher ANS intensity of the experimentally determined spectra. At pH 3, ANS (negatively charged probe) can easily bind to the positively charged BLG and the protonated sides of the PGM in addition to the hydrophobic binding. Moreover, this electrostatic interaction between ANS and the proteins can facilitate refolding of the proteins at low pH [37]. Accordingly, the unfolded terminal regions of PGM at pH 3 [15,34] may undergo folding again after binding with BLG, ANS or with BLG–ANS complex. Thus, ANS can be buried in the hydrophobic protein matrix. After all, it can be concluded that ANS can be buried within the PGM–BLG protein complex most effectively at pH 3 in addition to its higher electrostatic and hydrophobic binding capacity at pH 3.

Both intrinsic and extrinsic fluorescence spectroscopy results indicate that BLG has higher binding affinity to PGM rather than BSM, especially at pH 3. This may be correlated with the zeta-potential of mucins at varying pH. BSM is more negatively charged than PGM at all pH values due to the more abundant presence of negatively charged residues [15,38]. In fact, zeta potential of PGM is close to zero at pH 3. Reduction of the pH results in the protonation of negatively charged sialic and carboxylic acid residues [39], with consequent changes in the tertiary structure of the mucins, so that the hydrophobic regions are exposed. This behavior is characteristic for PGM than BSM. Near-UV CD spectroscopy results also support the changes in tertiary structure of PGM at pH 3 (Figure 5) while no significant change was observed for BSM [4]. It is likely that BSM still preserves its extended conformation at pH 3 due to the repulsion promoted by negatively charged residues. After all, it is anticipated that a hydrophobic interaction is the dominating interaction mechanism between BLG and PGM, whereas an electrostatic interaction is the dominating one for BLG and BSM at pH 3.

As the molecular-level interactions of food proteins and salivary proteins are critical to understand the textural, sensory, and nutritional properties of food products in digestion, the results of this study have great potential for understanding oral processing and digestion related applications of BLG. Further studies regarding the quenching potential of mucins would be valuable in order to assess a more detailed quenching mechanism of mucins on proteins.

## 3. Materials and Methods

BLG from bovine milk, BSM (Type I-S), and PGM (Type III) were purchased from Sigma Aldrich (Brøndby, Denmark), and were used as received. Protein solutions were prepared by dissolving proteins in 100 mM PBS solutions. By addition of HCl as appropriate, the pH values of the buffer solutions were adjusted to pH 7, 5, and 3 where the BLG is negatively, neutral, and positively charged, respectively [4]. All buffer solutions were filtered (polyethersulfone 0.20 mm).

### 3.1. Intrinsic Fluorescence Spectroscopy

All intrinsic fluorescence measurements were carried out using a FS5 Spectrofluorometer (Edinburgh Instruments, Livingston, UK) with a 150 W Xenon lamp and a single photon counting photomultiplier (PMT) detector (Hamamatsu, R928P). The excitation wavelength range (λ_ex_) was at 280 nm and the emission wavelength range was from 290 to 420 nm (measured every 2 nm). Other settings of the instrument were: a slit width of 2 nm (for both excitation and emission) and a photomultiplier (PMT) detector voltage of 1245 V. The protein solutions were scanned three times and the spectra were averaged to reduce the noise.

The quenching of BLG intrinsic fluorescence by different mucins was assayed. Tryptophan residues were used as intrinsic fluorophore. Since mucins absorb energy at the established emission wavelength [28], a blank was made for each mucin concentration, where the protein solution was replaced by phosphate buffered saline (PBS). The respective spectrum of each mucin was then subtracted from the emission spectrum of the corresponding mixture [28,33].

A dilution series of mucin solutions (0.2–10 μM) were prepared in PBS solutions at pH 7, 5, and 3. For each data point, 1 mL of the appropriate mucin solution was added into 1 mL BLG (56 μM) solution, to give a final mucin concentration in the range (0.1–5 μM) and a final BLG concentration of 28 μM. The change in fluorescence emission intensity was measured after 2 min of adding mucin to the BLG. The addition of a constant volume of quencher to the protein solution avoided complications due to dilution effects within titration type experiments. Each measurement was repeated in triplicate and the mean and standard deviation were calculated.

### 3.2. Extrinsic Fluorescence Spectroscopy

ANS fluorescence spectra were recorded at 25 °C using a Chirascan spectrophotometer (Applied Photophysics Ltd., Surrey, UK). The ANS spectra were recorded from 380 to 600 nm when λ_ex_ is 366.5 nm, using a 10 mm path length cuvette with a step size of 1 nm and a bandwidth of 1 nm. ANS was prepared as a 100 μM stock solution in a 10 mM PBS solution at pH 3, 5, and 7. The protein concentration was 1 mg/mL. The protein solutions were scanned three times and the spectra were averaged to reduce the noise. The spectra were obtained by subtracting the spectrum of the solvent (PBS buffer with ANS).

### 3.3. Circular Dichroism

The near-UV CD spectra were recorded at 25 °C using a Chirascan spectrophotometer (Applied Photophysics Ltd., Surrey, UK) from 450 to 260 nm using a 1 mm path-length cuvette with a step size of 2 nm and bandwidth of 0.8 nm. The protein solutions were scanned three times and the scans were averaged to reduce the noise. The spectra obtained were subtracted from the spectrum of the solvent.

## Data Availability

The study did not report any data.

## Sample Availability

Samples of the compounds are not available from the authors. All samples are commercially available.
